# Investigating the Potential Role of Capsaicin in Facilitating the Spread of Coxsackievirus B3 via Extracellular Vesicles

**DOI:** 10.3390/ijms27020661

**Published:** 2026-01-09

**Authors:** Shruti Chatterjee, Ramina Kordbacheh, Haylee Tilley, Devin Briordy, Richard T. Waldron, William D. Cutts, Jayden Aleman, Alexis Cook, Raeesa Dhanji, Lok-Yin Roy Wong, Stephen J. Pandol, Brandon J. Kim, DeLisa Fairweather, Jon Sin

**Affiliations:** 1 Department of Biological Sciences, University of Alabama, Tuscaloosa, AL 35401, USA; sc3118@njms.rutgers.edu (S.C.); ramina.kordbacheh@utdallas.edu (R.K.); hrtilley@crimson.ua.edu (H.T.); devinbriordy@gmail.com (D.B.); william.cutts@utdallas.edu (W.D.C.); aacook6@crimson.ua.edu (A.C.); brandon.kim@utdallas.edu (B.J.K.); 2 Department of Microbiology, Biochemistry and Molecular Genetics, Rutgers New Jersey Medical School, Newark, NJ 07103, USA; roy.wong@rutgers.edu; 3 Department of Biological Sciences, University of Texas at Dallas, Richardson, TX 75080, USA; jayden.aleman@utdallas.edu; 4 Department of Medicine, Cedars-Sinai Medical Center, Los Angeles, CA 90048, USA; richard.waldron@cshs.org (R.T.W.); stephen.pandol@cshs.org (S.J.P.); 5 College of Medicine, University of Arizona, Tucson, AZ 85724, USA; raeesadhanji@arizona.edu; 6 Department of Cardiovascular Medicine, Mayo Clinic, Jacksonville, FL 32224, USA; fairweather.delisa@mayo.edu

**Keywords:** coxsackievirus B3, TRPV1, capsaicin, mitophagy, HSP70, capsazepine, extracellular vesicles, pancreatitis

## Abstract

Coxsackievirus B3 (CVB3) is a picornavirus that causes systemic inflammatory diseases including myocarditis, pericarditis, pancreatitis, and meningoencephalitis. We have previously reported that CVB3 induces mitochondrial fission and mitophagy while inhibiting lysosomal degradation by blocking autophagosome-lysosome fusion. This promotes the release of virus-laden mitophagosomes from host cells as infectious extracellular vesicles (EVs), enabling non-lytic viral egress. Transient receptor potential vanilloid 1 (TRPV1), a heat and capsaicin-sensitive cation channel, regulates mitochondrial dynamics by inducing mitochondrial membrane depolarization and fission. In this study, we found that TRPV1 activation by capsaicin dramatically enhances CVB3 egress from host cells via EVs. Released EVs revealed increased levels of viral capsid protein VP1, mitochondrial protein TOM70, and fission protein phospho-DRP1. Moreover, these EVs were enriched in heat shock protein HSP70, suggesting its role in facilitating infectious EV release from cells. Furthermore, TRPV1 inhibition with capsazepine and SB-366791 significantly reduced viral infection in vitro. Our in vivo studies also found that SB-366791 significantly mitigates pancreatic damage and reduces viral titers in a mouse model of CVB3 pancreatitis. Given the lack of understanding regarding factors that contribute to diverse clinical manifestations of CVB3, our study highlights capsaicin and TRPV1 as potential exacerbating factors that facilitate CVB3 dissemination via mitophagy-derived EVs.

## 1. Introduction

Coxsackievirus is a positive-sense single-stranded RNA virus that belongs to the *Enterovirus* genus within the *Picornaviridae* family [1]. These non-enveloped viruses can infect a range of tissues, notably the central nervous system, heart, and pancreas, causing diseases from mild febrile illnesses to severe conditions like myocarditis, pericarditis, meningitis, and pancreatitis [2,3]. Their impact is particularly pronounced in neonates and young children; however, adults are also susceptible to these diseases [4,5]. Coxsackieviruses can be categorized into two primary groups: coxsackievirus A (CVA) and coxsackievirus B (CVB). CVA primarily targets the skin and mucous membranes, often causing conditions like hand, foot, and mouth disease (HFMD) and herpangina, while CVB is known to target internal organs, including the heart, pancreas, and central nervous system [6,7,8]. CVB can further be subdivided into six distinct serotypes, labeled CVB1 through CVB6, with CVB3 being particularly significant due to its impact on human health and its involvement in diverse clinical manifestations [9,10,11,12,13]. While CVB3 predominantly induces mild, self-limiting symptoms such as fever, rash, and upper respiratory tract illness, it can, albeit rarely, progress to severe systemic inflammatory diseases including pancreatitis, meningoencephalitis and myocarditis. The factors determining the clinical spectrum—from mild, cold-like symptoms to life-threatening complications—remain poorly understood. Further research is necessary to elucidate whether specific environmental factors may enhance viral pathogenicity and trigger the transition from mild to severe diseases.

Our previously published studies suggest that CVB3 can subvert mitophagy in host cells—a cellular degradation pathway by which damaged or dysfunctional mitochondria are packaged within autophagosomes that eventually become degraded via their fusion with acidic lysosomes [14,15,16,17]. A key process required for mitophagy is mitochondrial fission which removes damaged mitochondria from the healthy mitochondrial network [18]. In our earlier studies, we showed that CVB3 virions not only induce mitophagy but also become incorporated into mitophagosomes. Furthermore, others have shown that CVB3 can impair autophagosome-lysosome fusion, thus permitting virus-laden mitophagosomes to be released from the host cell as infectious extracellular vesicles (EVs) [19,20,21,22,23]. This mechanism serves as a non-lytic mode of viral dissemination that prolongs replication within host cells and enables them to evade host-neutralizing antibodies.

In this study, we seek to elucidate the potential involvement of transient receptor potential (TRP) channels in the induction of mitophagy and mitochondrial fission associated with CVB3 infection. Our previous work demonstrated that inhibition of the heat- and capsaicin-activated ion channel TRPV1 significantly attenuates CVB3 infection [22]. Moreover, like CVB3, TRPV1 activation has also been shown to induce mitochondrial membrane depolarization and subsequent mitochondrial fission [24,25,26]. Building upon these findings, our current study focuses on further exploring the involvement of TRPV1 in CVB3 pathogenesis. Given the convergence of CVB3-induced mitochondrial fission and TRPV1-mediated mitochondrial dynamics, we hypothesize that TRPV1 may serve as a critical intermediary facilitating enhanced CVB3 infection and dissemination.

One of the most well-known agonists of TRPV1 is capsaicin. Capsaicin, the active pungent compound found in chili peppers, is widely found in flavoring spices and plays a prominent role in the diets of numerous communities worldwide [24]. Beyond its culinary presence, capsaicin has been recognized for its various beneficial effects on human health, including its potential therapeutic roles in pain relief, inflammation, rheumatoid arthritis, vasomotor rhinitis and its well-documented anti-cancer properties [25,26,27,28,29]. In this study, we observed that activating TRPV1 with capsaicin significantly increased CVB3 infection in HeLa cervical cancer cells. This was mediated by dramatically increased viral shedding during capsaicin exposure. When interrogating the composition of released EVs, we not only saw significantly more viral capsid protein VP1 but also observed increased levels of mitochondrial protein TOM70 and mitochondrial fission protein phospho-DRP1 (Ser 616). These findings suggest the potential role of capsaicin in inducing mitophagy, which facilitates EV-based viral spread. In addition, capsaicin treatment further enhanced release of EVs enriched with heat shock protein HSP70. Numerous studies have demonstrated that HSP70 is a vital protein in the context of viral infections as it facilitates viral protein translation both at the initiation and elongation stages [30,31,32,33,34]. The heat shock response in cells is initiated by an increase in the fluidity of specific membrane domains and earlier studies have shown that plasma membrane-associated TRPV1 can directly trigger the membrane-dependent activation of HSPs [35,36]. Given that HSP70 has been identified as a potential biomarker of EVs shed from certain diseased cell types such as cancer cells [37,38,39], we hypothesize that capsaicin not only promotes mitophagy-based viral EV release, but more specifically facilitates the biogenesis of HSP70-positive viral EVs. This was further corroborated by silencing *HSPA1A*/HSP70 in HeLa cells, which led to a drastic reduction in viral protein concentration within EVs. Moreover, inhibiting HSP70 using VER-155008 significantly attenuated CVB3 infection. All these findings reveal how capsaicin-induced TRPV1 activation not only amplifies CVB3 infection but also highlights a novel mechanism of viral spread through HSP70-enriched EVs, unveiling new targets for therapeutic intervention. Furthermore, the action of TRPV1 was validated in vitro using the competitive antagonist capsazepine and in vivo using the allosteric inhibitor SB-366791 which not only demonstrated reduced viral infection, but also mitigated pancreatic viral burden, inflammation and damage.

Knowing that capsaicin exacerbates CVB3 infection as well as viral release via EVs, we hypothesize that this common dietary component may predispose to severe CVB3 infections. Due to the large gaps in our understanding of susceptibility factors that contribute to CVB3 diseases, we anticipate that this study will shed light on a very common dietary component that could potentially exacerbate CVB3 disease manifestation.

## 2. Results

### 2.1. Capsaicin Enhances CVB3 Infection

In 2020, our research identified a novel connection between TRPV1 and CVB3. We demonstrated that inhibition of TRPV1 using the potent and selective antagonist SB-366791 markedly suppresses CVB3 infection in vitro [22]. Based on these findings, we hypothesized that TRPV1 activation—via stimuli such as heat or capsaicin—may enhance viral infection by inducing mitochondrial depolarization, which in turn promotes mitochondrial fission. This process could not only support mitophagy but can also disrupt mitochondria-based antiviral responses [15,21,23].

**Figure 1:**
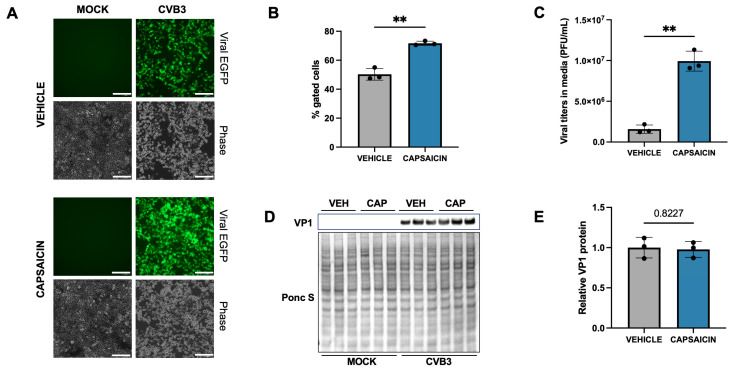
Capsaicin enhances CVB3 infection. Human cervical cancer cells HeLa were treated with 10 µM Capsaicin for 15 min and infected with EGFP-CVB3 at multiplicity of infection (MOI) of 0.001 for 48 h. (**A**). Fluorescence microscopy compares viral EGFP expression between vehicle (DMSO-treated) and capsaicin-treated cells. Phase contrast images are shown below respective fluorescence image fields. Scale bars represent 100 µm. (**B**). Flow cytometry of infected cells revealed significantly increased EGFP-positivity in capsaicin-treated cells as compared to controls. (**C**). Plaque assay quantification of infectious virus in extracellular media. (**D**). Western blot on cell lysates from (**A**) showing VP1 viral capsid protein. Ponceau S is shown below. (VEH: Vehicle; CAP: Capsaicin). (**E**). Densitometric quantification of Western blot in (**B**) (** *p* < 0.01; Student’s *t*-test, *n* = 3, values indicate mean ± standard deviation (SD)).

To investigate this hypothesis, we examined the effect of capsaicin, a known TRPV1 agonist, on CVB3 infection. Given that capsaicin concentrations in chili peppers range from 0.003 to 16.37 µM (calculated based on mg/kg capsaicin present in varying range of peppers), we selected a dose of 10 μM for our experiments. Additionally, several studies have used capsaicin in this concentration range, further supporting our choice for this investigation [40,41,42,43,44,45,46,47]. HeLa cells were treated with 10 μM capsaicin for 15 min before infecting them with enhanced green fluorescent protein (EGFP)-expressing-CVB3 (EGFP-CVB3) at a multiplicity of infection (MOI) of 0.001. A treatment window of 15 min was chosen based on prior literature which shows TRPV1 undergoes rapid agonist- and Ca^2+^-dependent desensitization shortly after capsaicin exposure. The study by Sanz-Salvador et al. shows that persistent capsaicin stimulation leads to functional desensitization of TRPV1 within minutes, followed by trafficking of the receptor toward lysosomal degradation. Based on these findings, we selected a 15 min treatment window to achieve strong TRPV1 activation while avoiding the later phases of severe desensitization and receptor loss [48]. Capsaicin treatment did not induce any cytotoxicity in HeLa cells as observed in LDH assay (Figure S1). Following infection for 48 h, a substantial enhancement of viral EGFP expression was observed in capsaicin-treated cells using fluorescence microscopy and flow cytometry (Figure 1A,B and Figure S2). This was further confirmed by plaque assay which revealed significantly higher viral titers in the extracellular media of capsaicin-treated cells (Figure 1C). However, Western blot analysis of the corresponding cell lysates revealed no significant differences in intracellular viral capsid protein VP1 levels between capsaicin-treated and vehicle-treated infected samples (Figure 1D,E). This discrepancy between Western blot and plaque assay suggests that following infection for 48 h, capsaicin may potentially induce viral egress from host cells, resulting in higher viral titers in media compared to cellular VP1 expression.

Additionally, comprehensive plaque assays conducted on both intracellular and extracellular fractions revealed that capsaicin treatment did not significantly affect the quantity of virus retained within the cells, while the amount of virus released into the extracellular media was markedly increased in treated cells compared to controls, highlighting capsaicin’s specific role in enhancing viral egress (Figure S3).

### 2.2. Capsaicin Induces Viral Egress and EV-Mediated Spread of CVB3

Recent studies, including our own, have shown that non-enveloped viruses such as CVB3 can spread intercellularly via EVs [15,23,49,50]. This unconventional strategy of viral egress offers several advantages: it enables the transport of multiple virions within a single vesicle, shields viral particles from neutralizing antibodies, and may extend the window of productive infection by bypassing immune detection [15,21,23].

**Figure 2:**
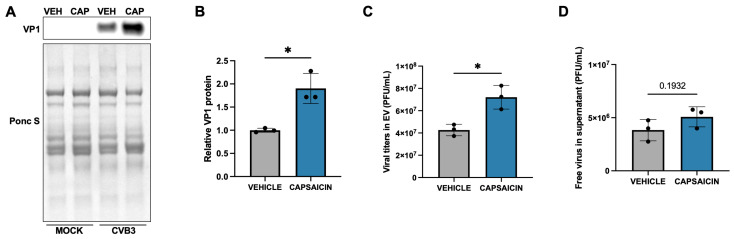
Capsaicin induces viral release in EVs. HeLa cells were treated with 10 µM Capsaicin for 15 min and infected with EGFP-CVB3 at an MOI of 0.001 for 48 h. (**A**). Western blot on isolated EVs showing VP1 viral capsid protein. Ponceau S is shown below. (VEH: Vehicle; CAP: Capsaicin) (**B**). Densitometric quantification of Western blot in (**A**) (* *p* < 0.05; Student’s *t*-test, *n* = 3). (**C**). Plaque assay quantification of viral titers in EV (* *p* < 0.05; Student’s *t*-test, *n* = 3) (**D**). Plaque assay of remaining free virus in the supernatant following EV isolation from capsaicin and DMSO-treated cells (*p* = 0.193; Student’s *t*-test, *n* = 3, values indicate mean ± SD).

To explore whether capsaicin influences this mode of viral dissemination, we treated HeLa cells with capsaicin, followed by infection with CVB3. After 48 h, EVs were isolated from the culture supernatant and analyzed. Western blotting revealed that EVs derived from capsaicin-treated cells contained significantly higher levels of the viral capsid protein VP1 compared to those from vehicle-treated controls (Figure 2A,B), suggesting an increase in EV-associated viral content. To further assess the infectivity of these vesicles, we performed plaque assays on both the isolated EVs and the remaining EV-depleted supernatants. Remarkably, the EVs from capsaicin-treated cells demonstrated significantly higher viral titers than those from control cells (Figure 2C). In contrast, the levels of free virus in the supernatant were not significantly different between the two groups (Figure 2D).

These results collectively suggest that capsaicin does not merely affect viral release in general; rather it specifically promotes the packaging and release of infectious CVB3 particles via EVs. This may represent a novel mechanism by which TRPV1 activation facilitates more efficient viral spread while potentially helping the virus evade host immune responses.

### 2.3. Capsaicin Increases Mitochondrial Content in EVs

**Figure 3:**
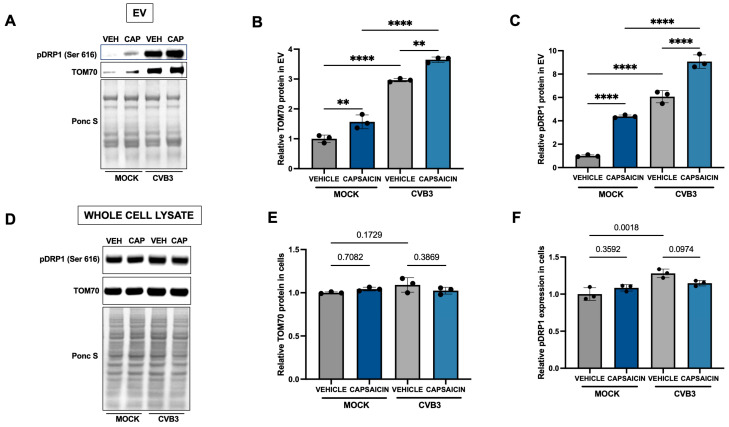
Capsaicin increases mitochondrial content in EVs. HeLa cells were treated with 10 µM Capsaicin for 15 min and infected with EGFP-CVB3 at multiplicity of infection (MOI) of 0.001 for 48 h. Western blot on EVs. (**A**) and whole cell lysates (**D**) detecting TOM70 and phospho-DRP-1 (Ser 616). Ponceau S is shown below. (VEH: Vehicle; CAP: Capsaicin). Densitometric quantification of Western blots for EVs (**B**,**C**) and cell lysates (**E**,**F**). (** *p* < 0.01; **** *p* < 0.0001; Student’s *t*-test, *n* = 3, values indicate mean ± SD).

To further investigate the origin of these EVs, we analyzed them for mitochondrial markers. HeLa cells were pretreated with 10 μM capsaicin for 15 min before infection with EGFP-CVB3 at an MOI of 0.001. After 48 h, we isolated EVs and performed Western blot analysis to examine the mitochondrial protein TOM70 and mitochondrial fission protein phospho-DRP1 (Ser 616) (Figure 3A–C). As expected, CVB3 infection increased the levels of these proteins in EVs. However, capsaicin treatment by itself also led to a significant increase in phospho-DRP1 (Ser 616) and TOM70 levels within EVs, implying that the TRPV1 agonist promotes mitochondrial fission and contributes to the presence of mitochondrial fragments within EVs. Furthermore, combining capsaicin treatment with infection further enhanced the expression of these proteins, albeit modestly. Interestingly, corresponding analysis of whole-cell lysates showed no significant changes in the intracellular levels of these proteins following either treatment or infection (Figure 3D–F), indicating that the observed effects are EV-specific. To assess EV purity, expression of the exosomal marker ALIX was also measured (Figure S4A,B).

Together, these findings suggest that capsaicin facilitates CVB3 dissemination, at least in part, by promoting the release of mitophagosome-enriched EVs containing mitochondrial fragments and fission-related proteins.

### 2.4. Capsaicin-Induced Infectious EVs Are Enriched with HSP70

The 70 kDa heat shock protein (HSP70) is a key component of the cellular chaperone network, crucial for managing various cellular stresses [51] and has been considered to be a biomarker for tumor-derived exosomes [52,53,54]. Moreover, HSP70 expression can be triggered due to an increase in the fluidity of specific membrane domains in plasma membrane, and TRPV1, being a key regulator in cellular thermosensory pathway, plays a crucial role in inducing membrane-dependent HSPs [35,36]. While HSP70 functions primarily to support proper protein folding and maintain cellular homeostasis, it can also be co-opted by viruses to aid in viral protein folding and survival under adverse host conditions [55,56,57,58]. Specifically, in the context of CVB3, HSP70 has been shown to facilitate viral protein translation at both the initiation and elongation stages [30]. HSP70 has also been implicated in the induction of mitophagy through its interaction with PTEN-induced kinase 1 (PINK1) in HEK 293 cells [59]. Moreover, we have observed that the Middle East Respiratory Syndrome Coronavirus ORF8b (MERS-CoV ORF8B) impedes HSP70-IKKε interactions and attenuates type I interferon responses [60].

**Figure 4:**
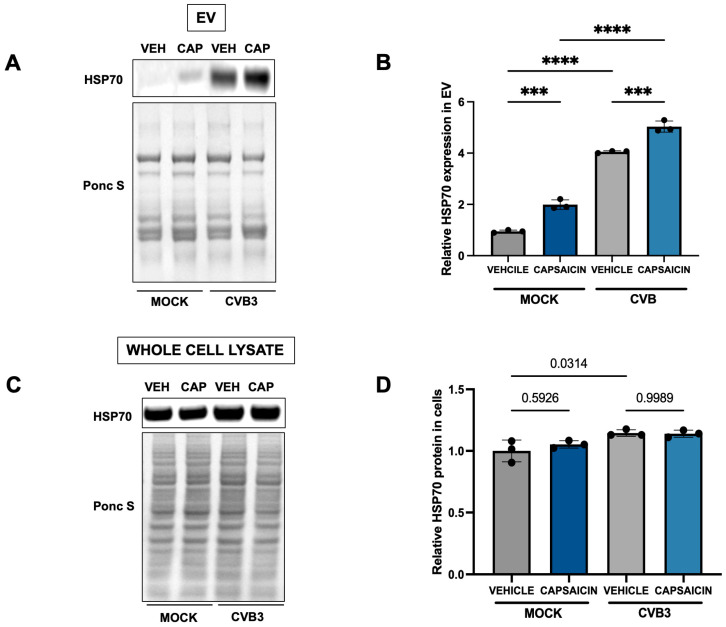
Capsaicin induced EVs are enriched with HSP70. HeLa cells were treated with 10 µM Capsaicin for 15 min and infected with EGFP-CVB3 at MOI of 0.001 for 48 h. (**A**). Western blot on EVs and (**C**) whole cell lysates detecting HSP70. Ponceau S is shown below. (VEH: Vehicle; CAP: Capsaicin) (**B**,**D**). Densitometric quantification of Western blot in (**A**,**C**). (*** *p* < 0.001, **** *p* < 0.0001; Student’s *t*-test, *n* = 3, values indicate mean ± SD).

Given these roles, we investigated whether HSP70 contributes to the release of CVB3-containing EVs in capsaicin-treated HeLa cells. Consistent with previous experiments, capsaicin-treated cells were infected with EGFP-CVB3 at an MOI of 0.001 for 48 h. EVs were then isolated and analyzed for HSP70 content by Western blotting. As observed in Figure 4, infected EVs shows higher expression of HSP70 relative to mock, likely reflecting involvement of HSP70 in infection. Moreover, the combined effect of capsaicin and infection also resulted in a modest yet significant increase in HSP70 (Figure 4A,B). Whereas analysis of whole-cell lysates revealed only minor changes in HSP70 expression following either capsaicin treatment or CVB3 infection (Figure 4C,D). This indicates that the observed upregulation in HSP70 expression is specifically for EVs.

Collectively, these findings suggest that capsaicin potentially promotes the release of HSP70-enriched EVs and raise the possibility that HSP70 plays a previously unrecognized role in EV-mediated viral egress.

### 2.5. Targeting HSP70 Limits CVB3 Dissemination via EVs

To further ascertain the involvement of HSP70 in the dissemination of CVB3 via EVs, we performed siRNA-mediated knockdown of *HSPA1A*, the gene encoding HSP70. HeLa cells were transfected with *HSPA1A*-targeting siRNA, and after 48 h, were infected with EGFP-CVB3 at an MOI of 0.001. EVs and cell lysates were collected 24 h post-infection for subsequent analyses, including Western blotting to assess HSP70 and VP1 levels.

**Figure 5:**
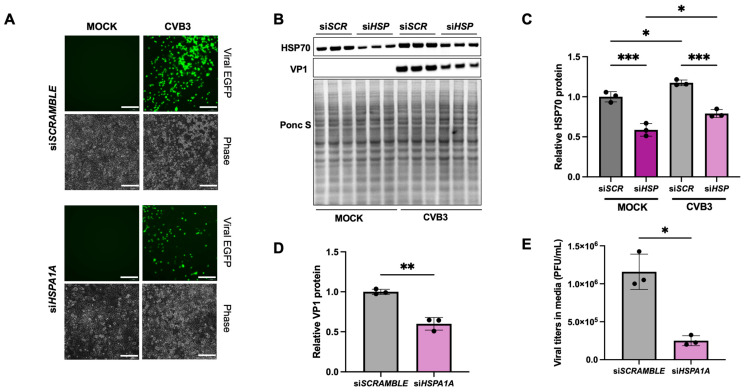
Silencing *HSPA1A* inhibits CVB3 infection. (**A**). HeLa cells were transfected with siRNA targeting HSP70 (*siHSPA1A*) or scrambled RNA (*siSCRAMBLE*) for 48 h and subsequently infected with EGFP-CVB3 at MOI 0.001 for 24 h. (**A**) Fluorescence microscopy comparing viral EGFP expression between cells transfected with either *siSCRAMBLE* or *siHSPA1A*. Corresponding phase contrast images are shown below the fluorescence images. Scale bars represent 150 µm. (**B**). Western blot on cell lysates from (**A**) detecting HSP70 and VP1 viral protein levels. Ponceau S is shown below. (SCR: *SCRAMBLE*; HSP: *HSPA1A*) (**C**,**D**). Densitometric quantification of Western blots in (**B**). (**E**). Plaque assay quantification of infectious virus in media from *siSCRAMBLE* and *siHSPA1A* transfected cells. (* *p* < 0.05, ** *p* < 0.01, *** *p* < 0.001; Student’s *t*-test, *n* = 3, values indicate mean ± SD).

Although *HSPA1A* silencing resulted in a modest reduction in intracellular HSP70 expression (Figure 5B,C), the functional consequences were more striking. Fluorescence microscopy, Western blot analysis of cell lysates, and plaque assays of the extracellular media all showed a substantial decrease in viral presence in *HSPA1A*-depleted cells (Figure 5A–E), indicating that even partial knockdown of HSP70 significantly impairs CVB3 infection. Notably, extracellular viral titers were significantly reduced, suggesting that *HSPA1A* silencing impacts viral release and potentially reduces the release of infectious EVs; however, this may simply be a reflection of reduced infection overall. To investigate this further, we analyzed VP1 expression in isolated EVs and observed a significant reduction in viral protein content in EVs derived from *HSPA1A*-silenced cells compared to controls (Figure S5A–C).

**Figure 6:**
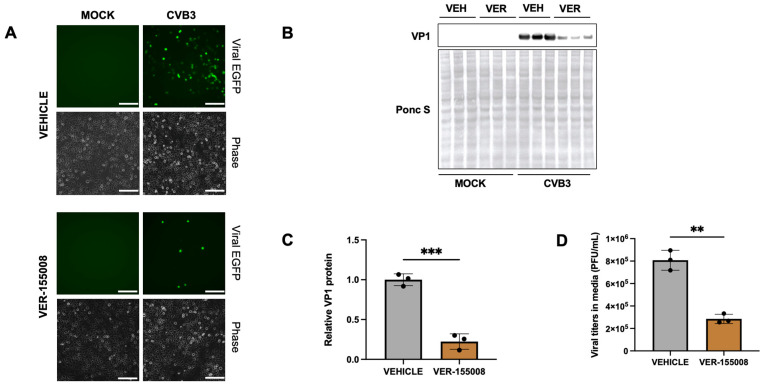
HSP70 inhibitor VER-155008 inhibits CVB3 infection. HeLa cells were treated with 20 µM VER-155008 for 1 h and infected with EGFP-CVB3 at multiplicity of infection (MOI) of 0.001 for 24 h. (**A**). Fluorescence microscopy compares viral EGFP expression between vehicle (DMSO-treated) and VER-treated cells. Phase contrast images are shown below respective fluorescence image fields. Scale bars represent 100 µm. (**B**). Western blot on cell lysates from (**A**) showing viral capsid protein VP1. Ponceau S is shown below. (VEH: Vehicle; VER: VER-155008). (**C**). Densitometric quantification of Western blot in (**B**). (**D**). Plaque assay quantification of infectious virus in media from cells in (**A**). (** *p* < 0.01, *** *p* < 0.001; Student’s *t*-test, *n* = 3, values indicate mean ± SD).

Moreover, the involvement of HSP70 was further confirmed by treating cells with HSP70 inhibitor VER-155008. HeLa cells were treated with 20 µM VER-155008 for 1 h prior to infecting with EGFP-CVB at MOI of 0.001. After 24 h of infection, Western blot analysis and plaque assays demonstrated a significant reduction in both viral VP1 protein levels and extracellular viral titers in inhibitor-treated cells compared to controls (Figure 6A–D). Lactate dehydrogenase (LDH) assays also confirmed that the treatment did not induce cytotoxicity under these conditions (Figure S6).

Taken together, all these findings reinforce our hypothesis that HSP70 plays a crucial role in the release of infectious EVs from CVB3-infected cells and capsaicin enhances viral spread, at least in part, by promoting the secretion of HSP70-enriched EVs. Whether these EVs also originate from mitophagosomes or represent a distinct vesicular trafficking pathway that parallels mitophagy remains an open question. Nonetheless, the data highlight a critical role for HSP70 in mediating EV-based viral dissemination.

### 2.6. Capsazepine and SB-366791 Inhibit CVB3 Infection

**Figure 7:**
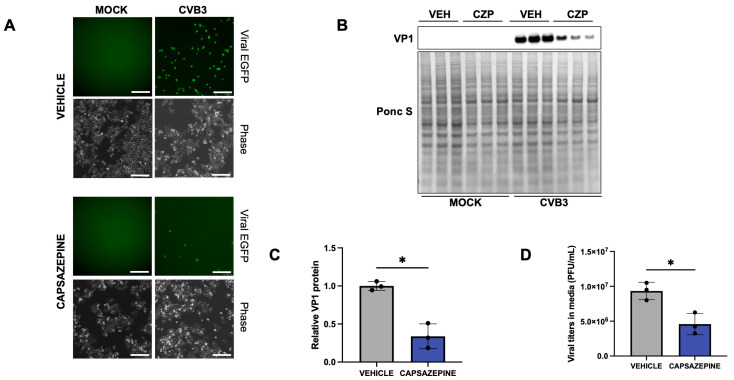
Capsazepine inhibits CVB3 infection. HeLa cells were treated with 40 µM capsazepine for 4 h and infected with EGFP-CVB3 at multiplicity of infection (MOI) of 0.001 for 48 h. (**A**). Fluorescence microscopy compares viral EGFP expression between vehicle (DMSO-treated) and capsazepine-treated cells. Phase contrast images are shown below respective fluorescence image fields. Scale bars represent 100 µm. (**B**). Western blot on cell lysates from (**A**) showing VP1 viral capsid protein. Ponceau S is shown below. (VEH: Vehicle; CZP: Capsazepine). (**C**). Densitometric quantification of Western blot in (**B**,**D**). Plaque assay quantification of infectious virus in media from cells in (**A**). (* *p* < 0.05; Student’s *t*-test, *n* = 3, values indicate mean ± SD).

Given that capsaicin-mediated TRPV1 activation enhanced CVB3 infection, we next investigated whether pharmacological inhibition of TRPV1 could mitigate viral replication and release in vitro. Among available TRPV1 antagonists, capsazepine was one of the first identified and has been widely used to explore TRP channels as therapeutic targets, particularly in pain management, without notable adverse effects [61,62,63]. For this study, HeLa cells were pretreated with 40 µM capsazepine for 4 h prior to infection with CVB3 at an MOI of 0.001. After 48 h, Western blot analysis revealed a significant reduction in intracellular VP1 levels, while plaque assays showed a corresponding decrease in viral titers in the extracellular media (Figure 7A–D). The treatment did not induce any cytotoxicity in HeLa cells as observed in LDH assay (Figure S7). Despite its effectiveness, capsazepine is a thiourea derivative with limited specificity; it can non-selectively interact with other voltage-gated ion channels and nicotinic acetylcholine receptors, which may confound interpretation. SB-366791, a cinnamide analogue, is a TRPV1 inhibitor known for its high selectivity [64]. Our previous work demonstrated the antiviral effects of SB-366791 on CVB3-infected HeLa cells [22]. To further confirm the involvement of TRPV1 in CVB3 infection, HeLa cells were pre-treated with SB-366791 prior to infection with CVB3 for 48 h at an MOI of 0.001. Treatment with SB-366791 significantly attenuated viral infection, as demonstrated by reduced viral protein levels in cell lysates and lower viral titers in the extracellular media (Figure S8). Similar to capsazepine, SB-366791 treatment did not induce cytotoxicity, as confirmed by LDH assays (Figure S9).

### 2.7. SB-366791 Significantly Reduces CVB3 Infection In Vivo

**Figure 8:**
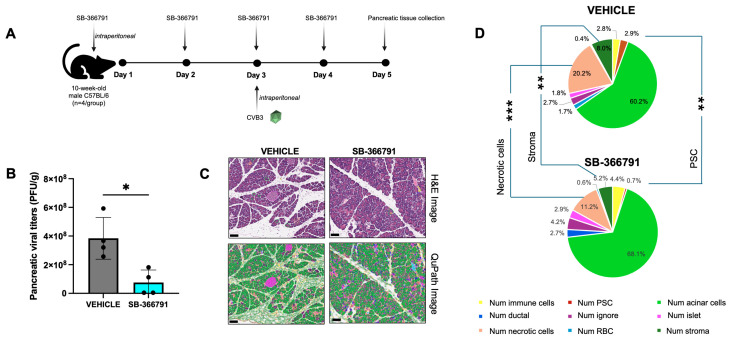
SB-366791 reduces pancreatic viral titers and tissue destruction in CVB3-infected mice. (**A**). Experimental timeline showing 10-week-old C57BL/6 male mice were treated with 1 mg/kg SB-366791 or equivalent volume vehicle each day for 3 days via intraperitoneal (IP) administration. On the second day of treatment, mice were infected IP with EGFP-CVB3 at a concentration of 10^7^ PFU/mouse. A total of 2 days post-infection, mice were sacrificed, and pancreata were collected. (**B**). Pancreatic viral titers as measured by plaque assays on pancreatic homogenates. (* *p* < 0.05; Student’s *t*-test, *n* = 4). (**C**). Representative microscopic images of the pancreas stained with hematoxylin and eosin (H&E, **left panels**; scale bar: 100 µm) or QuPath images (**right panels**; scale bar: 100 µm) for automatic quantification of cell types and stroma of same images shown in the **left panels**. (**D**). Percentage of each cell type feature detected in vehicle (**upper chart**) and SB-366791 (**lower chart**) treated infected mice. The percentage of each cell type was determined in QuPath by dividing the number of cells assigned to each classified subtype by the total number of cells detected within the corresponding tissue segment. Data are presented as pie charts (mean%). (** *p* < 0.005, *** *p* < 0.001; Student’s *t*-test, *n* = 4).

Given that capsaicin-mediated TRPV1 activation increased infection, we sought to determine whether blocking TRPV1 would affect CVB3 infection in vivo. Here, we investigated the effects of SB-366791 in a mouse model of CVB3 pancreatitis. Male C57BL/6 mice were administered 1 mg/kg SB-366791 or an equivalent volume of vehicle intraperitoneally for three consecutive days. One day after the initial treatment, mice were infected intraperitoneally with EGFP-CVB3 at a dose of 107 PFU. Two days post-infection, the pancreata were harvested to perform plaque assays and histology (Figure 8A). Throughout the treatment and infection period, the mice showed no adverse symptoms, such as reduced feeding or grooming. Plaque assay of pancreatic tissues revealed that SB-366791 significantly reduced viral titers in the pancreas (Figure 8B). Hematoxylin and eosin staining showed severe tissue damage in vehicle-treated infected mice characterized by extensive immune cell infiltration, pancreatic edema, and necrosis. In contrast, while SB-366791-treated mice also exhibited pancreatic damage, the severity was notably reduced compared to the vehicle controls (Figure 8C and Figure S10). Furthermore, SB-366791-treated pancreatic tissues showed significant reductions in the number of necrotic cells (*p* = 0.0002), stromal cells (*p* = 0.0036) and pancreatic stellate cells (PSCs) (*p* = 0.0010), which are the primary effector cells in the process of fibrosis, a key pathological characteristic in pancreatic diseases such as chronic pancreatitis and pancreatic cancer (Figure 8D) [65,66]. These findings support our in vitro results and underscore the role of TRPV1 in modulating CVB3 infection in host cells.

## 3. Discussion

In the past few years, most studies have highlighted the role of TRPV1 in regulating irritant-induced airway responses and the dissemination of airborne viral particles, particularly for respiratory viruses such as rhinovirus, respiratory syncytial virus, and measles virus [67]. However, few studies have investigated the link between TRP channels and CVB3 infection. Our recent study demonstrated that the cold and menthol-sensitive transient receptor potential channel TRPM8 plays a crucial role in inhibiting mitochondrial fission (a prerequisite for mitophagy) during CVB3 infection [22]. In contrast, the role of heat-sensing ion channel TRPV1 in CVB3 infection remains understudied.

In the present study, we found that activating TRPV1 with the commonly consumed spice compound capsaicin significantly enhances CVB3 infection in HeLa cells. In addition, it markedly increases the release of CVB3-laden EVs, which have the potential to intrinsically enhance subsequent rounds of infection in neighboring cells. For many years, the *Picornaviridae* family of naked viruses were thought to escape from the host cell exclusively via cytolysis [68,69,70,71]. This mode of dissemination releases free naked virions, making them susceptible to host-neutralizing antibodies. However, since 2013, several studies have shown that certain non-enveloped viruses can utilize EVs for dissemination, thus evading host-neutralizing antibodies [21,23,72,73,74,75,76,77]. It has been shown that CVB3 can escape host cells by packing multiple virions in phosphatidylserine-enriched membrane-enclosed EVs, thus facilitating the transfer of a high number of viral quasi-species, which can eventually infect and propagate viral progeny to neighboring cells more efficiently than free viruses [15,49].

Upon investigating the composition of EVs released after capsaicin treatment, we observed a marked increase in the viral capsid protein VP1, as well as elevated expression of the mitochondrial protein TOM70 and the mitochondrial fission protein phospho-DRP1 (Ser 616). These findings suggest that capsaicin-mediated TRPV1 activation may promote mitophagy to aid in the release of viral particles through EVs that contain mitochondrial components. In addition to these mitochondrial markers, we also observed a significant increase in the expression of HSP70 in the released EVs. The heat shock response is a highly conserved cellular response that is initiated by an increase in the fluidity of specific membrane domains, thus triggering the activation of heat-shock gene expression to safeguard and restore labile proteins and membranes. In a previous study, TRPV1 was demonstrated to act as a key regulator of the cellular thermosensory pathway, leading to membrane-dependent activation of HSPs [35]. Our findings reveal that TRPV1 agonist capsaicin enhances HSP70 expression levels that exclusively increase in EVs, rather than in the cells. This suggests a previously undescribed mechanism in which HSP70 may play a pivotal role in promoting the dissemination of infectious EVs among host cells.

Earlier studies have already highlighted the role of HSP70 in supporting CVB3 infection. This is largely due to the presence of an IRES region within the 5′UTR of the *HSPA1A* mRNA, similar to CVB3 genomic RNA, which allows it to be translated during CVB3 infection even when the cap-dependent translation is impaired [30,78]. In our study, silencing *HSPA1A* in HeLa cells resulted in a marked reduction of CVB3 infection, particularly in EVs, confirming the chaperone’s role in facilitating CVB3 spread via EVs. However, the precise role of HSP70 in the induction of mitophagy-derived EVs requires further investigation. Previous studies have linked HSP70 to both cellular autophagy and PINK1-mediated mitophagy, and HSP70 inhibition has been demonstrated to influence mitochondrial dynamics in the absence of mitophagy [59,79]. Thus, inhibiting HSP70 in our study may have impeded the mitophagy process, which in turn reduced the release of infectious EVs. These findings may open new avenues for the development of antiviral therapies aimed at disrupting CVB3 transmission via EVs. Furthermore, it remains unclear exactly how the virus interacts with or influences players in this TRPV1/HSP70 pathway of EV biogenesis.

We observed that inhibiting TRPV1 with specific TRPV1 antagonist SB-366791 reduces infection in vivo. Earlier studies that have implicated TRPV1 in the context of pancreatitis have shown that TRPV1 levels are elevated in non-infectious animal models of pancreatitis [80,81]. This alludes to the fact that activation of pancreatic TRPV1-positive nociceptive neurons contributes to neurogenic pancreatic inflammation. In our study, we corroborated this by finding that treating mice with TRPV1 inhibitor SB-366791 markedly attenuates CVB3 titers and tissue destruction in the pancreas. While we did not directly test TRPV1 activation via capsaicin in vivo, our results support the idea that TRPV1 may be a promising antiviral therapeutic target. Future studies could investigate whether TRPV1 activation, for example through chronic exposure of mice to physiologically relevant, commonly consumed concentrations of capsaicin, exacerbates viral infection outcomes, thereby providing complementary insight into the role of TRPV1 in vivo.

**Figure 9:**
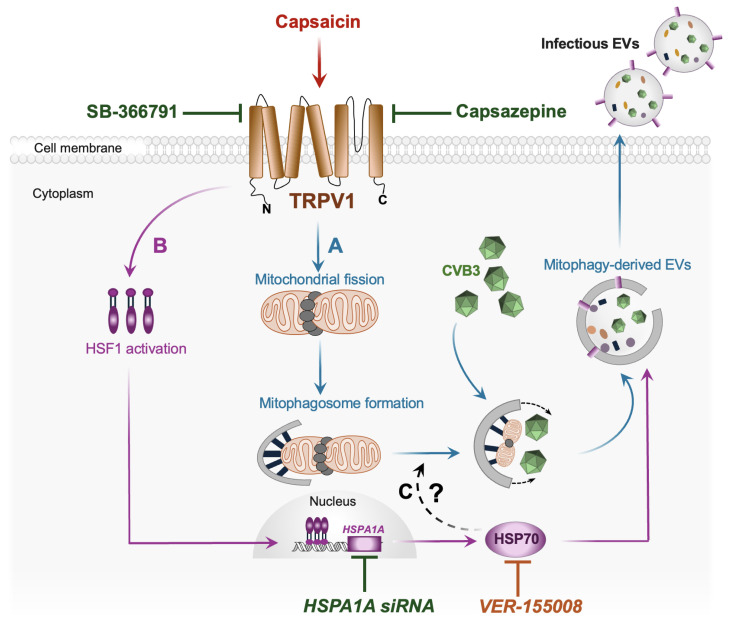
Capsaicin has a significant impact on CVB3 infection dynamics. (A). Capsaicin-mediated TRPV1 activation induces mitochondrial fission, which leads to mitophagy. CVB3 hijacks these mitophagosomes to form mitophagosome-derived EVs, within which the virus multiplies. These EVs then facilitate viral dissemination, serving as a vehicle for CVB3 release. (B). In addition, these capsaicin-treated EVs also showed higher levels of HSP70. Earlier literature suggests that TRPV1 plays a crucial role in controlling the cellular heat shock response via activation of Heat shock Factor-1 (HSF-1) [35]. HSF-1 then trimerizes and translocate to the nucleus where it binds to *HSPA1A* promotor, resulting in translation of the protein HSP70 [82]. In our study, we observed that inhibiting HSP70 significantly impaired CVB3 release via EVs, indicating that HSP70 is crucial for viral egress. (C). Previous studies have shown a prominent role of HSP70 in mitophagy, particularly in HEK293 cells, but whether HSP70 interacts with mitochondrial proteins or primes cells for mitophagy in the context of CVB3 infection remains to be fully explored and needs further investigation.

In summary, our findings reveal a novel mechanism by which capsaicin enhances the spread of CVB3. Capsaicin-mediated TRPV1 activation induces mitochondrial fission, subsequently promoting mitophagy. CVB3 appears to hijack these mitophagosomes to generate mitophagosome-derived EVs, within which the virus can propagate. These EVs then facilitate viral dissemination, serving as a vehicle for CVB3 release while also aiding in immune evasion. Notably, we observed an upregulation of heat shock protein HSP70 in capsaicin-treated EVs, suggesting a functional role for HSP70 in the release of infectious viral EVs (Figure 9). Silencing or pharmacological inhibition of HSP70 significantly impaired CVB3 release, highlighting its importance in viral egress. Although previous studies have identified a role for HSP70 in mitophagy, particularly in HEK293 cells, whether HSP70 directly interacts with mitochondrial proteins or primes cells for mitophagy during CVB3 infection remains to be determined. These findings provide valuable new insights into how host cellular pathways can be co-opted by the virus to promote dissemination and immune evasion.

A potential limitation of this study is the purity of the isolated EVs. While our findings support the role of EVs in CVB3 dissemination, it remains possible that a subset of the isolated vesicles may still harbor membrane-associated, non-EV-bound virus. This could confound interpretation of EV-specific viral release. In addition, the widely-used polymer-based precipitation method including ExoQuick, may co-isolate small amounts of cellular debris. Therefore, while the detection of mitochondrial proteins TOM70 and p-DRP1 in our EV preparations suggests their association with EVs, minor contamination cannot be completely excluded. However, previous studies using similar isolation protocols have demonstrated undetectable levels of non-EV proteins in EV fractions, including nuclear and ER markers supporting [21]. Still, future studies could improve EV purity by incorporating nanoparticle tracking analysis, electron microscopy, or other optimizations into the EV purification protocol. Studies exposing mice chronically to a commonly consumed capsaicin concentration may help elucidate whether dietary capsaicin may influence CVB3 disease outcomes.

The fact that capsaicin is a widely consumed dietary compound raises important questions about how common dietary components could modulate viral pathogenesis and contribute to variability in disease outcomes. Our data suggest that TRPV1 activation, through dietary exposure or pharmacological means, may significantly impact CVB3 infection dynamics. The identification of TRPV1 as a modulator of CVB3 spread offers promising therapeutic potential for mitigating severe disease manifestations, particularly through targeted TRPV1 inhibition. As we move forward, it will be crucial to explore how dietary interventions or TRPV1-targeted therapies could be leveraged to reduce viral burden and improve clinical outcomes in CVB3 and potentially other viral infections associated with EV-mediated transmission.

## 4. Materials and Methods

### 4.1. Cell Culture

HeLa RW cells derived from human cervical cancer cells (originally obtained from Rainer Wessly, University of San Diego, La Jolla, CA, USA) were maintained in Dulbecco’s modified Eagle’s medium (Sigma-Aldrich, St. Louis, MO, USA; D6429) supplemented with 10% FBS (Avantor, Radnor Township, PA, USA; 89510-166) and antibiotic/antimycotic solution (Sigma-Aldrich; A5955). All chemicals used were of the highest grade available.

### 4.2. Treatments

HeLa cells were treated with TPRV1 agonist capsaicin (Sigma-Aldrich; M2028), TRPV1 antagonists capsazepine (Sigma-Aldrich; C191), SB-366791 (Sigma-Aldrich; S0441), as well as HSP70 inhibitor VER-155008 (Millipore Sigma, Burlington, MA, USA; SML02171). All these chemicals were dissolved in DMSO. Cells were treated with 10 μM capsaicin for 15 min, 40 μM capsazepine for 4 h, 10 μM SB-366791 for 24 h and 20 μM VER-155008 for 1 h prior to CVB3 infection.

### 4.3. Generation of CVB3 Stocks

CVB3 stocks were generated from the pMSK1 plasmid that was generously gifted by Dr. Ralph Feuer at the San Diego State University, CA, USA. Recombinant CVB3 expressing enhanced green fluorescent protein (EGFP-CVB3) was prepared following a protocol described previously [83]. In order to generate the pMSK1 plasmid, HeLa RW cells were transfected with plasmid pH3, that encodes a myocarditic strain of CVB3 [70,84]. A unique SfiI restriction site was then added to the backbone of this viral clone. The resulting plasmid, now known as pMSK1, can facilitate the insertion of foreign DNA fragments of interest into CVB3 genome, resulting in subsequent isolation of recombinant coxsackievirus after its transfection into HeLa cells [81]. The DNA sequence encoding enhanced green fluorescent protein (EGFP) was amplified from plasmid pEGFP and cloned into pMSK1 to generate recombinant EGFP-CVB plasmid. HeLa RW cells were subsequently transfected with the EGFP-CVB construct using Lipofectamine 2000 (Thermo Fisher Scientific, Waltham, MA, USA; 11668027). After 48 h, cells were harvested, and freeze fractured thrice to release virions. Free-thawed cells were then centrifuged at 600× *g* for 10 min. The supernatants were collected and considered ‘passage 1’ viral stocks, which were subsequently infected in fresh HeLa cells to generate ‘passage 2’ viral stocks. Concentrations of the newly prepared viral stocks were then determined by plaque assay.

### 4.4. CVB3 Infection of Host Cells

HeLa cells were infected with EGFP-CVB3 at a multiplicity of infection (MOI) of 0.001. Cells were inoculated with frozen viral stocks whose viral load had been calculated by plaque assay. Equivalent amounts of DMEM growth media were added to the corresponding controls, commonly referred to as mock infected cells. After 48 h of infection, mock and CVB3-infected cells were imaged using a Nikon Ti2 inverted epifluorescence microscope that is equipped with a Qi2 camera (Nikon, Tokyo, Japan) and an NiS Elements software version AR.5.30.05.

### 4.5. Lactate Dehydrogenase Cytotoxicity Assay

Lactate dehydrogenase (LDH) release was measured to assess cytotoxicity following treatment with capsazepine, SB-366791 and VER-155008. The assay was performed using the LDH Activity Assay Kit (Sigma-Aldrich; MAK464-1KT) according to the manufacturer’s instructions. Briefly, HeLa cells were treated with 40 µM capsazepine, and culture supernatants were collected and stored at −80 °C until further analysis. For the assay, duplicate samples were processed in a 96-well plate, with each well receiving a mixture of the supplied MTT solution, NAD solution, diaphorase enzyme, substrate buffer, and cell supernatant. The optical density (OD) of each well was measured at 565 nm at two time points: immediately after reagent addition (0 min) and following 25 min of incubation at room temperature. Absorbance readings were obtained using a Varioskan™ LUX multimode microplate reader (Thermo Fisher Scientific, Waltham, MA, USA). LDH activity was calculated as the change in absorbance over time and used to evaluate potential cytotoxic effects of capsazepine treatment.

### 4.6. Cell Lysis and Western Blot

Following treatment and infection, all cells were lysed using chilled RIPA buffer containing 50 mM Tris-HCl (Sigma-Aldrich; 10812846001), 1% NP-40 (Sigma-Aldrich; 74385), 0.5% sodium deoxycholate (Sigma-Aldrich; D6750), 0.1% sodium dodecyl sulfate (Sigma-Aldrich; L3771), 150 mM sodium chloride (Sigma-Aldrich; 13423), 2 mM EDTA (ethylenediaminetetraacetic acid) (Sigma-Aldrich; E9884), protease inhibitor cocktail (Thermo Fisher Scientific; A32965) and PhosSTOP phosphatase inhibitors (Sigma-Aldrich; 4906845001). Cells were then mechanically disrupted, and cell slurries were centrifuged at 15,000× *g* for 10 min at 4 °C and supernatants were collected to measure the protein concentrations using bicinchoninic acid solution (Thermo Fisher Scientific; 23228). Laemmli sample buffer containing 1% bromophenol blue (Allied Chemical, Morristown, NJ, USA; 0332), 1.5 M Tris-Cl pH 6.8, glycerol (Sigma-Aldrich; G5516), and β-mercaptoethanol (Thermo Fisher Scientific; BP176-100) was then added to equal amounts of protein and then loaded onto 4–12% Bis-Tris protein gels. Proteins were then transferred to nitrocellulose membranes, and membranes were stained with Ponceau S solution (Sigma-Aldrich; SLCQ5486) prior to blocking with 3% bovine serum albumin (BSA) (Sigma-Aldrich; A9647) in Tris-buffered saline with Tween 20 (TBS-T) (Sigma-Aldrich; P1379). After blocking at room temperature for one hour, the membranes were incubated with primary antibody solutions diluted in 3% Bovine Serum Albumin and TBS-T. Primary antibodies used in this study were as follows: VP1 (Mediagnost, Reutlingen, Germany; Cox mAB 31A2, 1:2000), TOM70 (Proteintech, Rosemont, IL, USA; 14528-1-AP, 1:1000), phospho-DRP1 (Ser 616) (Cell Signaling Technology, Danvers, MA, USA; 3455, 1:1000), HSPA1A (Thermo Fisher Scientific; PA5-34772, 1:5000) and ALIX (Proteintech; 12422-1-AP, 1:5000). Following overnight incubation, membranes were washed in TBS-T and incubated in anti-mouse secondary antibody (Sigma-Aldrich; 12-349, 1:3000) and anti-rabbit secondary antibody (Sigma-Aldrich; F9887, 1:3000). Following incubation for 1 h, membranes were washed in TBS-T and imaged with SuperSignal West Dura Extended Duration chemiluminescent substrate (Thermo Fisher Scientific; 34075) via iBright FL1500 Imaging System (Thermo Fisher Scientific) and Azure 600 Western Blot Imaging System (Azure Biosystems, Dublin, CA, USA). Densitometry was performed using ImageJ software (The National Institutes of Health (NIH), https://imagej.net/ij/, version: 1.54a, Access date: 20 January 2023) with background subtraction applied to all quantifications.

### 4.7. Plaque Assay

Following infection with CVB3, the viral titers in extracellular media were calculated using plaque assay. For this, HeLa cells were initially grown to confluency in 6 well plates. After 48 h, media was aspirated and 400 µL of serially diluted extracellular media (from infected samples) were added to the cell monolayer. Cells were then incubated for 1 h with occasional shaking at an interval of 15 min. Following this, infected cells were overlaid with a 50:50 mixture of 1.2% molten agar combined with 2× DMEM. The plates were incubated at 37 °C for 48 h and agar plugs were subsequently fixed with plaque fixing solution containing 25% acetic acid and 75% methanol for 30 min. After removing the plugs, the fixed cells were stained with 2.34% crystal violet solution for one hour, following which the plaques were counted.

### 4.8. Flow Cytometry

HeLa cells were treated with 10 μM capsaicin for 15 min prior to CVB3 infection for 48 h at MOI of 0.001. The supernatants from infected cells were individually collected in 15 mL conical tubes. The cells were then washed with phosphate-buffered saline (PBS) which was also collected in corresponding tubes to ensure collection of already detached cells. Adherent cells were then detached with Trypsin-EDTA (Thermo Fisher Scientific; 25200056) and subsequently collected in respective tubes. Following this, 10 mL growth medium was added to each tube and centrifuged at 500× *g* for 10 min. The supernatant was discarded, and cells were resuspended and fixed in 5 mL 4% formaldehyde. After fixation for 15 min, cells were centrifuged at 500× *g* for 10 min. Supernatant was discarded and cells were washed by resuspending in 10 mL PBS and centrifuging at 500× *g* for 10 min. Supernatant was discarded and cells were resuspended in 500 µL fresh PBS. Cells were then filtered in filter tubes to ensure single-cell suspension and analyzed via Attune NxT flow cytometer (Thermo Fisher Scientific, Waltham, MA, USA).

### 4.9. Isolation of Extracellular Vesicles

EVs were isolated from HeLa cells using the Exoquick-TC polymer-based exosome precipitation kit (System Biosciences, Palo Alto, CA, USA; EXOTC50A-1). Cells were seeded in 10 cm dish, treated with capsaicin, and then infected with eGFP-CVB3 for 48 h. One day post-infection, the supernatants were collected and centrifuged at 3000× *g* for 15 min to eliminate residual cell debris. Supernatants were then transferred to new conical tubes, with one-fifth volume of Exoquick-TC added to each one. The tubes were incubated overnight at 4 °C and EVs were pelleted by centrifugation at 1500× *g* for 30 min. Purified EV pellets were washed twice with 2 mL PBS and finally harvested in chilled RIPA buffer containing protease and phosphatase inhibitors. Concentrations of the EVs were measured by BCA assay and samples were subsequently prepared for Western blot analysis.

To determine whether this method can precipitate “free virus”, we performed this EV-precipitation method on viral stocks. Three independent viral stock vials were used. Following an initial high-speed centrifugation, supernatants were transferred to new tubes and processed using the above-mentioned Exoquick-based EV preparation protocol. After precipitation, supernatants were transferred to fresh tubes (mentioned as supernatant in Figure S11). Although no visible “pellet” was observed following the Exoquick treatment, the bottom of the tube was gently washed once with PBS and resuspended in an equal volume of media (mentioned as pellet in Figure S11). Plaque assays comparing viral titers in the supernatant versus the “pellet” showed that only a small amount of “free virus” is potentially precipitated (Figure S11).

### 4.10. siRNA Transfection

HeLa cells were seeded in 6 well plates and transfected with human *HSPA1A*-targeting siRNA (Santa Cruz Biotechnology, Dallas, TX, USA; sc-29352) using Effectene Transfection Reagent (Qiagen, Hilden, Germany; 301425). The transfection reagents were added following manufacturer’s guidelines. After 24 h, the media was replenished with fresh DMEM growth media and incubated further for 24 h before infection with CVB3 (MOI 0.001). EVs were then isolated from these cells and Western blots and plaque assays were subsequently performed.

### 4.11. Mouse Treatments

Animal ethics: All experiments involving mice were performed following the National Institutes of Health guidelines and were approved by the Institutional Animal Care & Use Committee of The University of Alabama. The animals were anesthetized using isoflurane and were sacrificed by performing cervical dislocation.

Treatment: Prior to treatment, SB-366791 was dissolved in DMSO at a concentration of 10 mM. Following this, the TRPV1 inhibitor was first injected intraperitoneally in 10-week-old male C57BL/6 mice at 1 mg/kg/day for 2 days. At the second day of treatment, mice were infected with EGFP-CVB3 (10^7^ PFU/mouse) via intraperitoneal injection. They were again treated with SB-366791 the following day and were sacrificed on second day post infection. The pancreata was harvested and pancreatic tissues were either flash frozen for plaque assays or fixed with 4% formaldehyde for histology. In order to perform plaque assay, the frozen pancreatic tissue was weighed and homogenized in DMEM using a TissueLyzer LT instrument (Qiagen). Homogenates were then centrifuged at 1000× *g* for 10 min at 4 °C and the supernatants were collected for plaque assay.

### 4.12. Statistics and Software

All statistical analyses were performed using GraphPad Prism version 10.2.1. The values in the experiments indicate mean ± standard deviation (SD). For pair-wise comparison, Student’s *t*-tests were performed, and for multiple comparisons, one-way ANOVA was performed. A *p* < 0.05 was accepted as statistical significance. (* *p* < 0.05, ** *p* < 0.01; *** *p* < 0.001; **** *p* < 0.0001). *p* values equal to or greater than 0.05 are not significant. CVB3-infected cells were imaged using a Nikon Ti2 inverted epifluorescence microscope equipped with a Qi2 camera (Nikon) and NiS Elements software version AR.5.30.05. Absorbance readings in LDH cytotoxicity assay were performed using a Varioskan™ LUX multimode microplate reader (Thermo Fisher Scientific). Western blots were imaged using iBright FL1500 Imaging System (Thermo Fisher Scientific) and Azure 600 Western Blot Imaging System (Azure Biosystems). Densitometry was performed using ImageJ software (The National Institutes of Health (NIH), https://imagej.net/ij/, Access date: 20 January 2023). For histology studies, H&E slides were scanned using the Aperio AT2 microscope slide scanner (Leica Biosystems, Deer Park, IL, USA) and imported into QuPath software (open source, version 0.2.1) [85].

## Data Availability

All data supporting the conclusions of this article are included within the article and its Supplementary Materials.

## Supplementary Materials

The following supporting information can be downloaded at: https://www.mdpi.com/article/10.3390/ijms27020661/s1.

## Abbreviations

The following abbreviations are used in this manuscript:

CVB3	coxsackievirus B3
EV	extracellular vesicle
TRPV1	transient receptor potential vanilloid 1
CVA	coxsackievirus A
CVB	coxsackievirus B
HFMD	hand, foot, mouth disease
TRP	transient receptor potential
EGFP	enhanced green fluorescent protein
EGFP-CVB3	enhanced green fluorescent protein-expressing coxsackievirus B3
MOI	multiplicity of infection
HSP70	70 kDa heat shock protein
PINK1	PTEN-induced kinase 1
MERS-CoV	Middle East respiratory syndrome coronavirus
LDH	lactate dehydrogenase
PSC	pancreatic stellate cell
Ponc S	Ponceau S
Veh	vehicle
Cap	capsaicin
Czp	capsazepine
*siSCR*	scrambled siRNA
*siHSP*	*HSPA1A*-targeting siRNA
VER	VER-155008
HSF1	heat shock factor 1
PFU	plaque forming units
