# Supplementary figures and images for: Investigating the Potential Role of Capsaicin in Facilitating the Spread of Coxsackievirus B3 via Extracellular Vesicles
Source: Int J Mol Sci. 2026 Jan 9;27(2):661. doi: 10.3390/ijms27020661 (PMC12841461; doi:10.3390/ijms27020661)

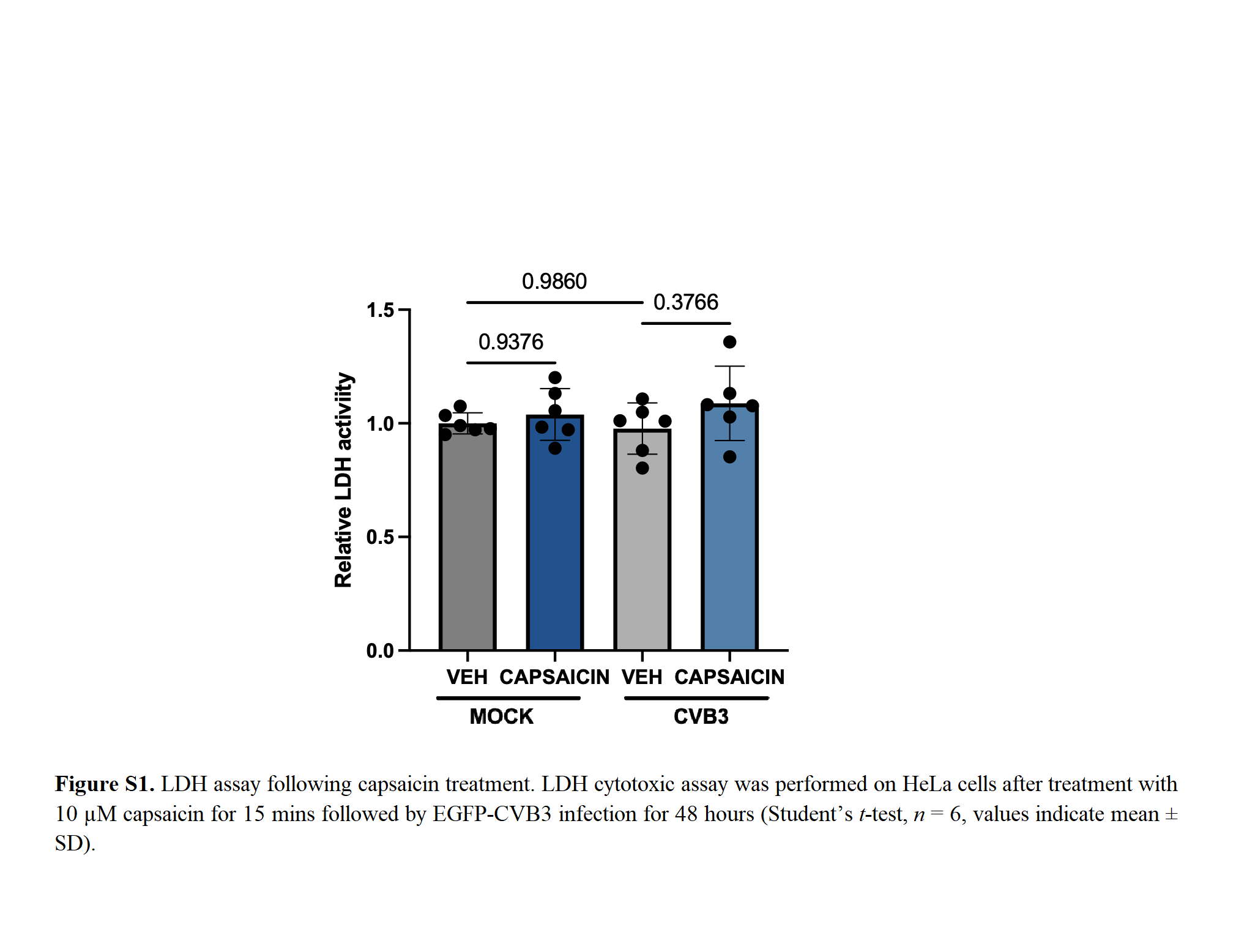

Supplement: Supplementary file 1 [file ijms-27-00661-s001.zip › Figure S1_IJMS.png]

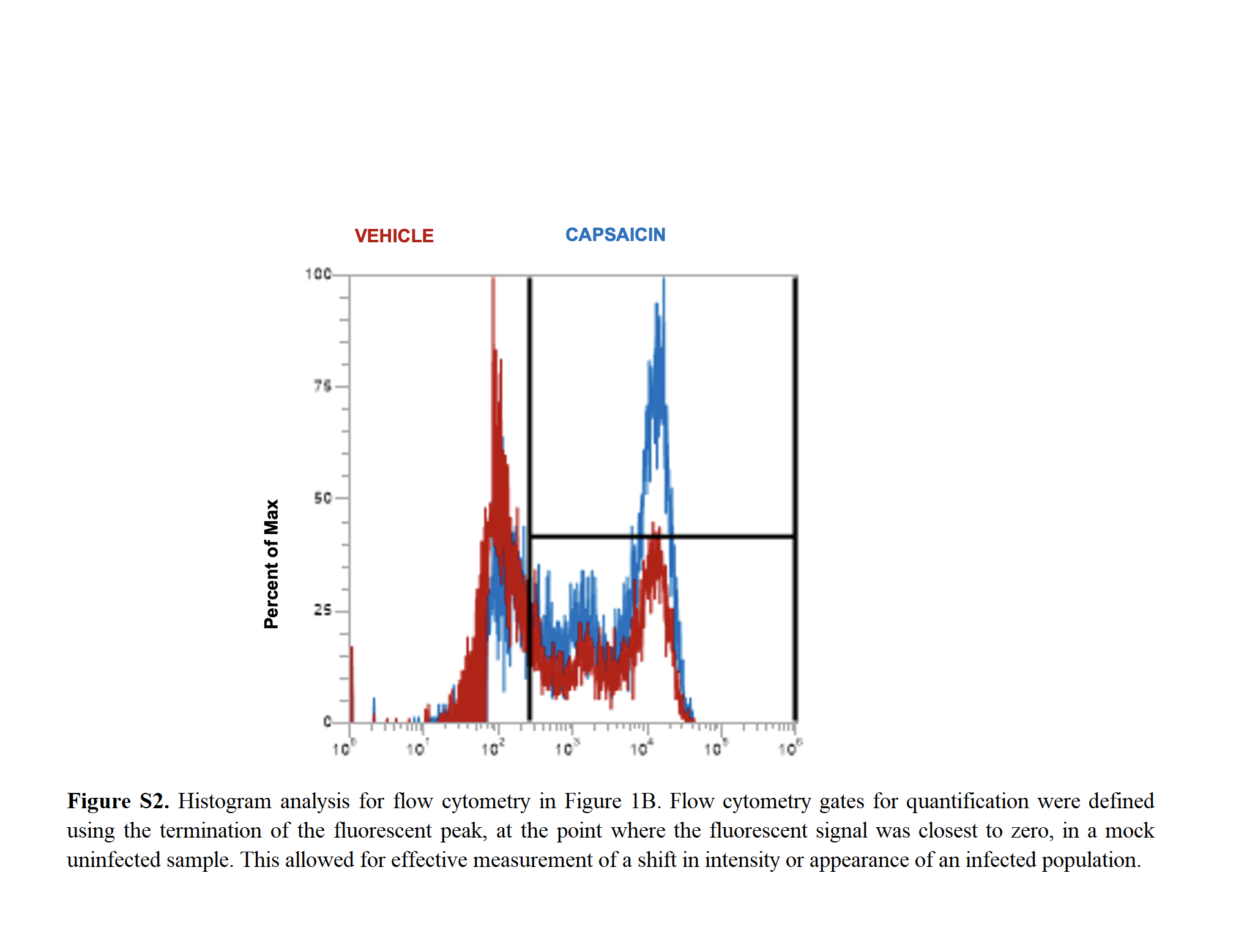

Supplement: Supplementary file 1 [file ijms-27-00661-s001.zip › Figure S2_IJMS.png]

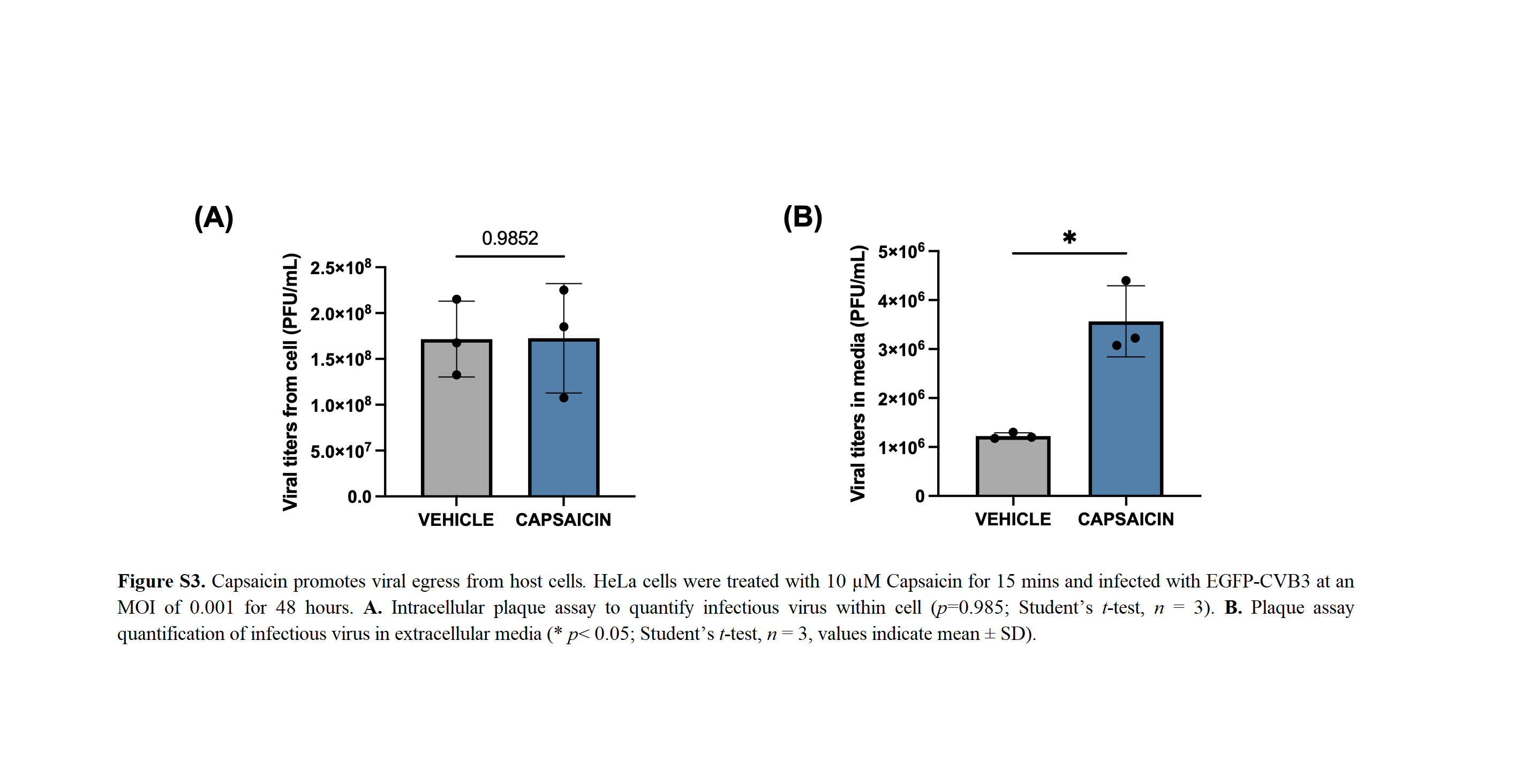

Supplement: Supplementary file 1 [file ijms-27-00661-s001.zip › Figure S3_Proofread.png]

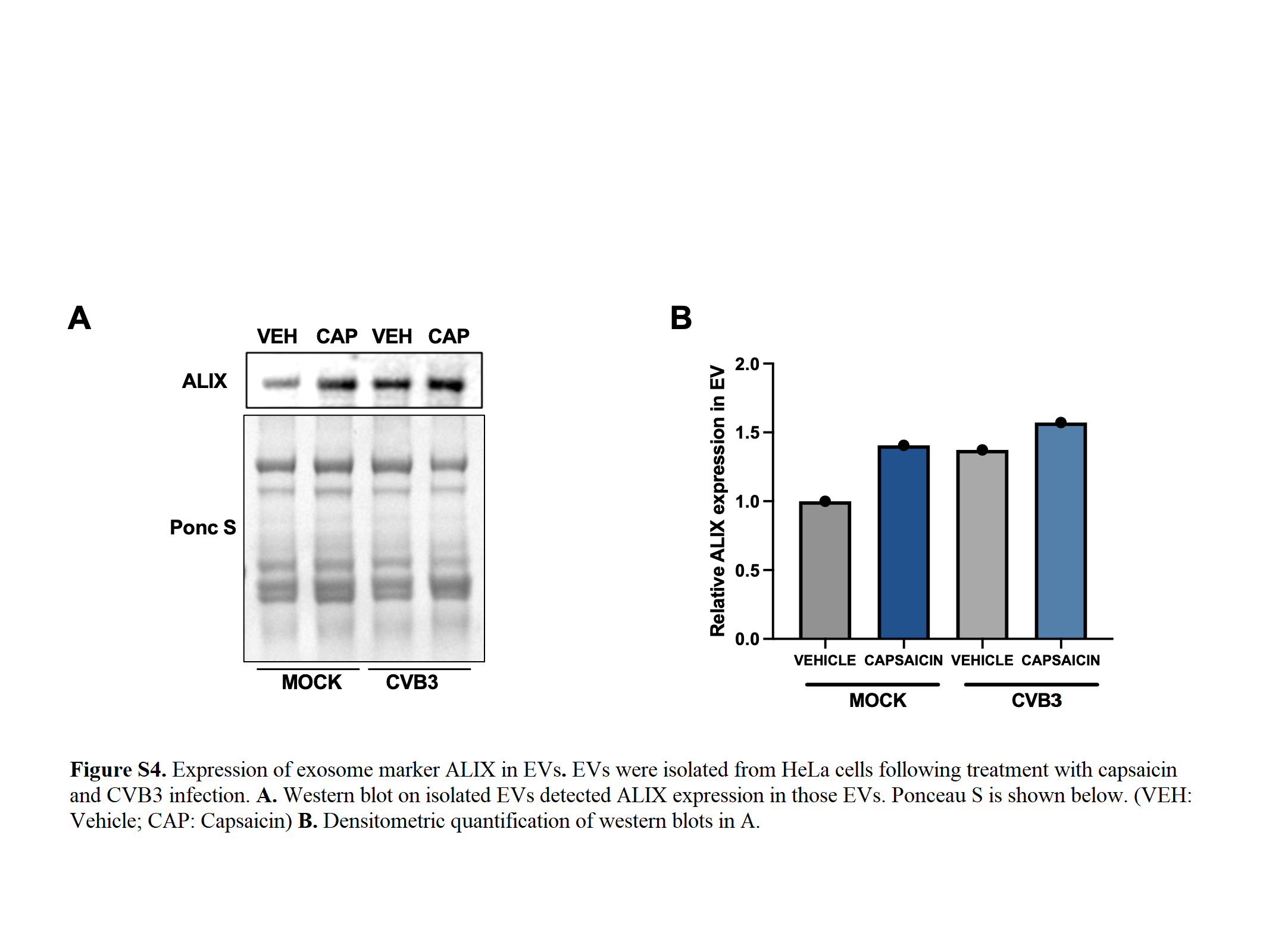

Supplement: Supplementary file 1 [file ijms-27-00661-s001.zip › Figure S4_IJMS.png]

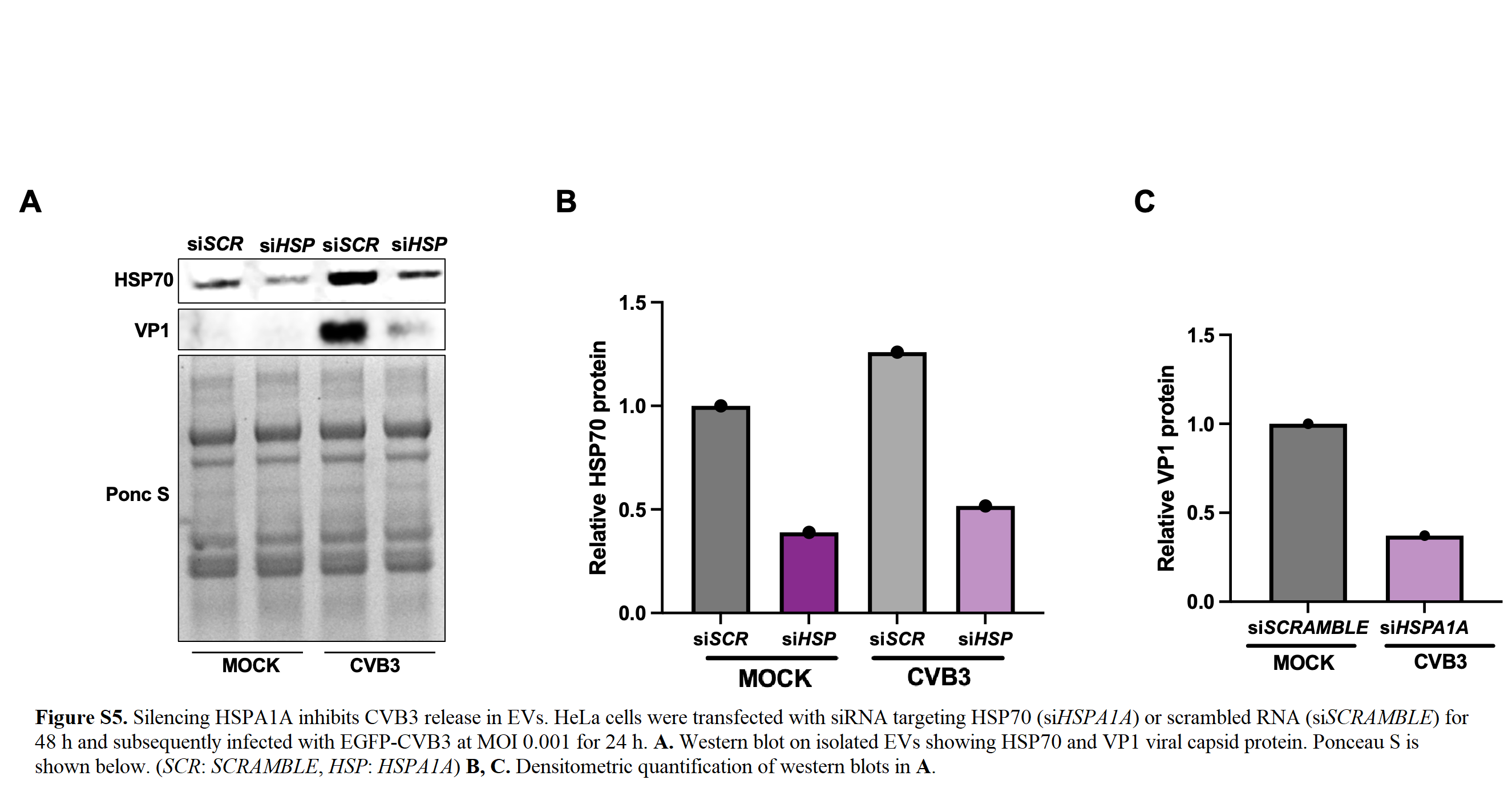

Supplement: Supplementary file 1 [file ijms-27-00661-s001.zip › Figure S5_IJMS.png]

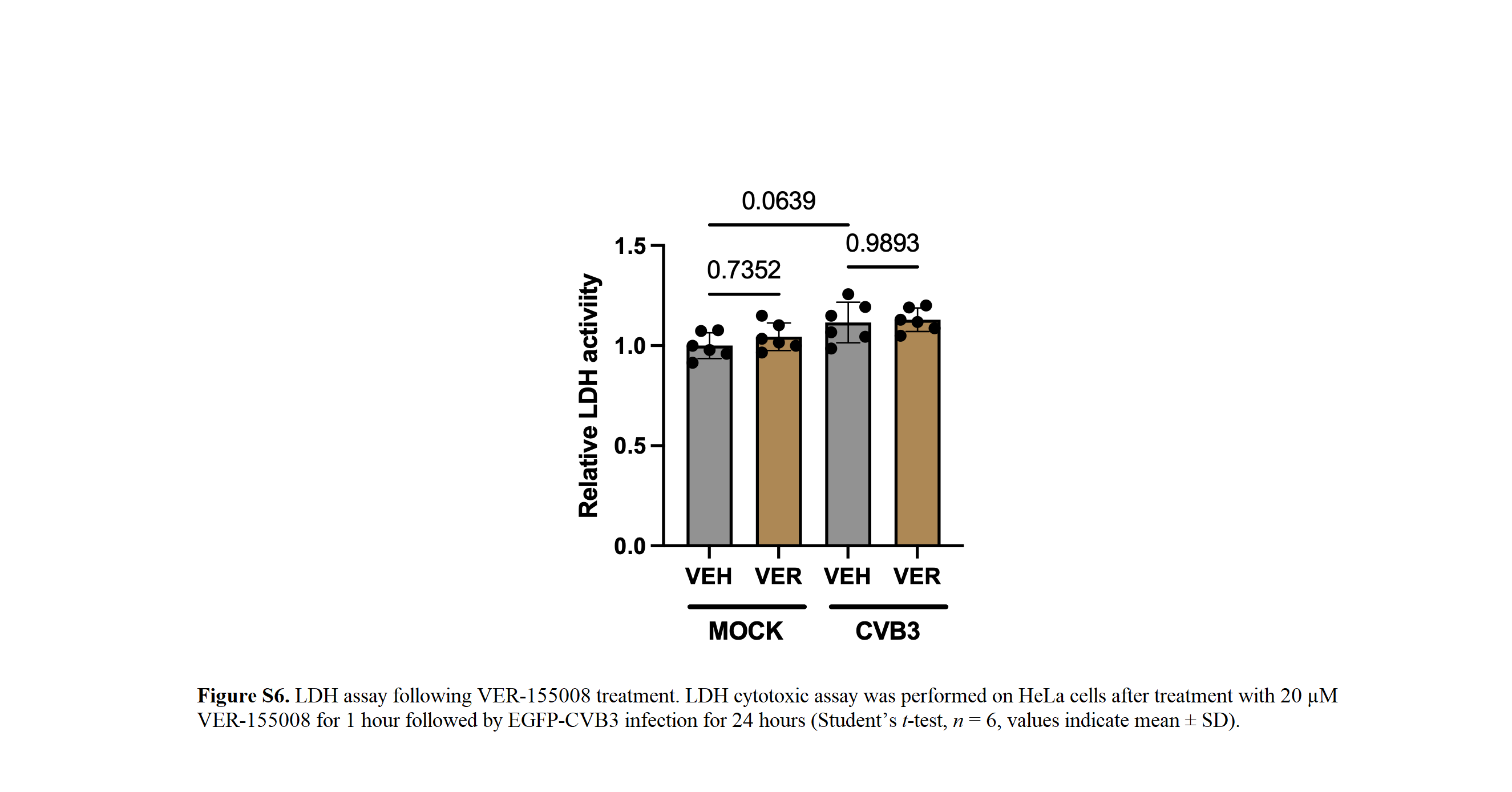

Supplement: Supplementary file 1 [file ijms-27-00661-s001.zip › Figure S6_IJMS.png]

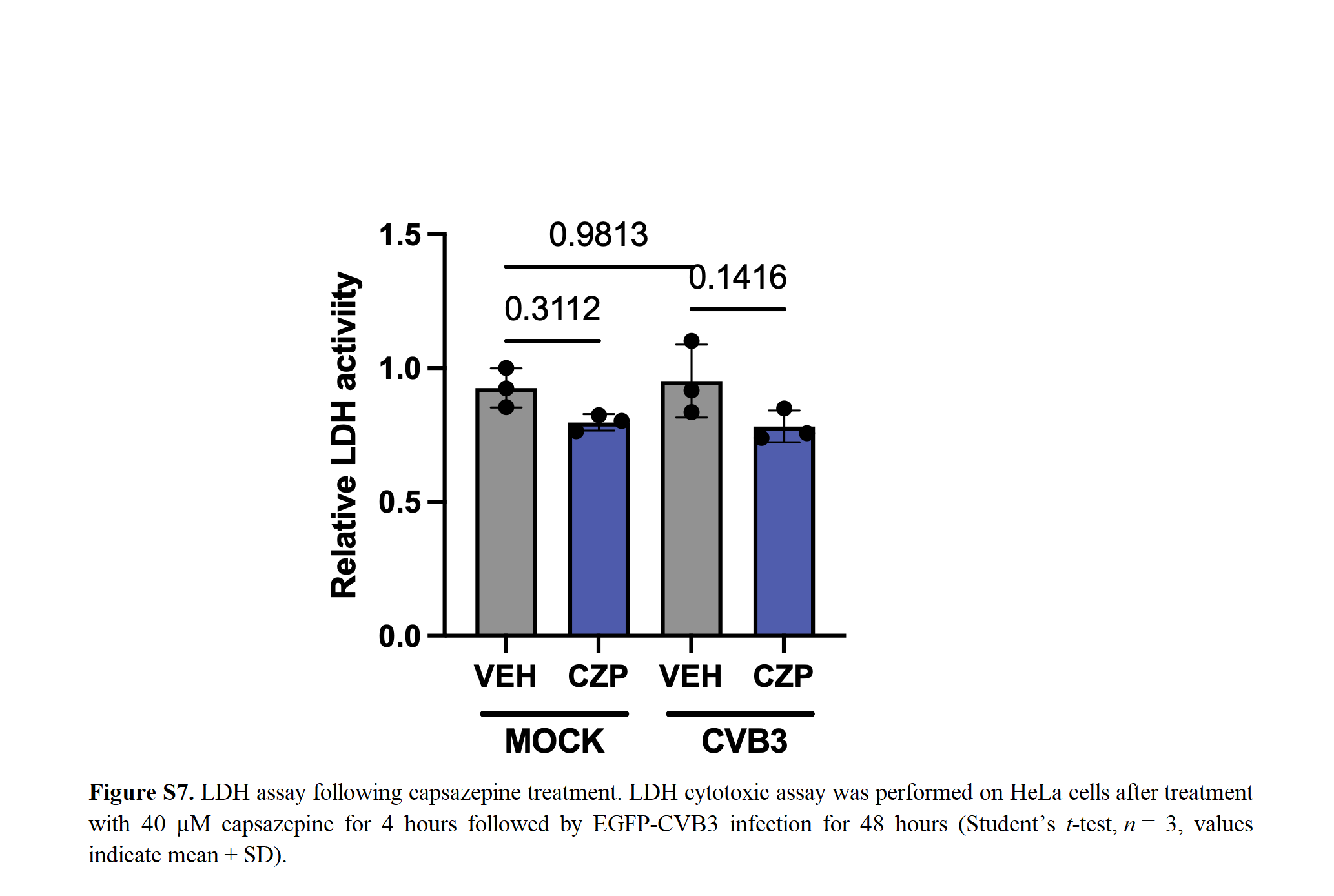

Supplement: Supplementary file 1 [file ijms-27-00661-s001.zip › Figure S7_IJMS.png]

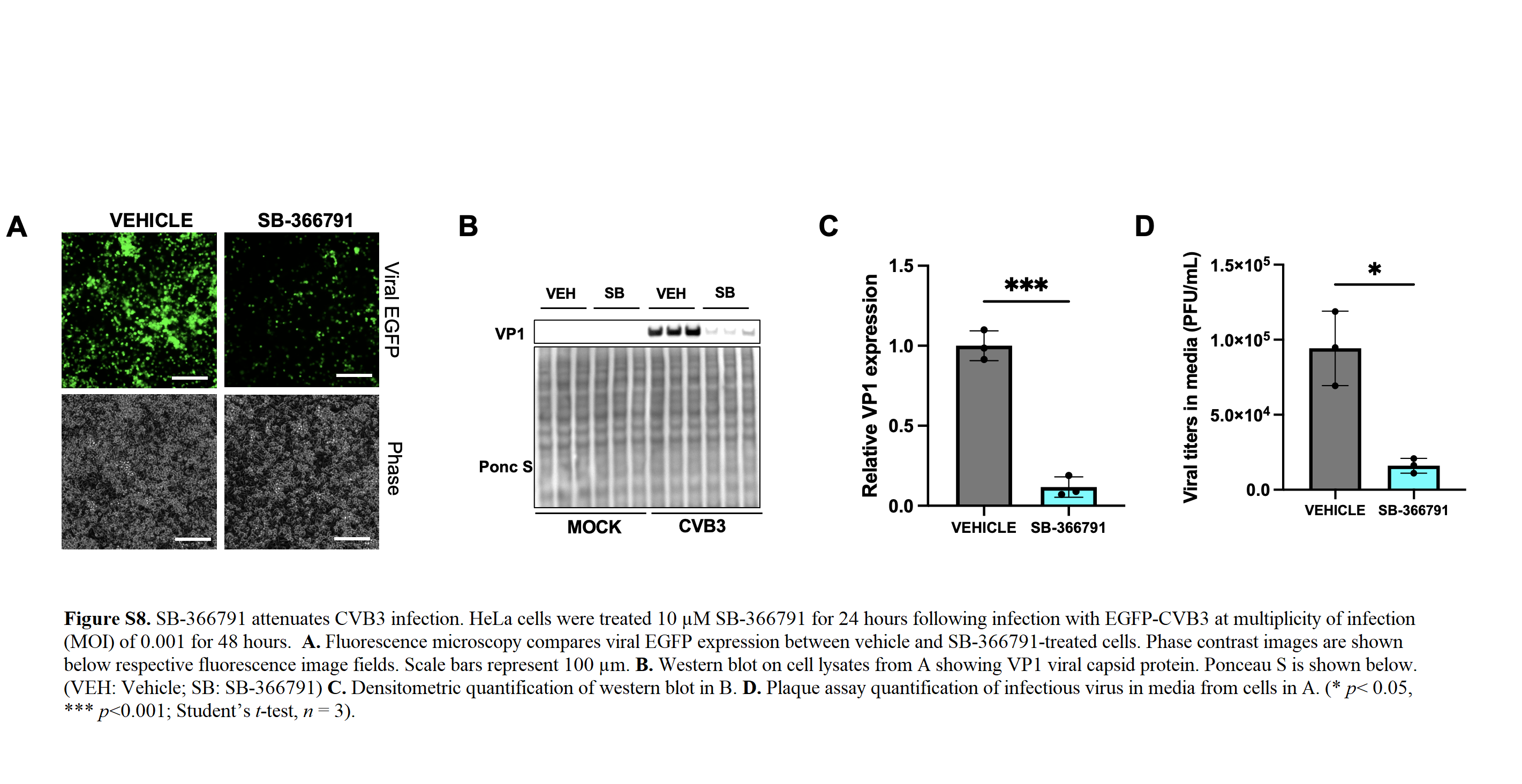

Supplement: Supplementary file 1 [file ijms-27-00661-s001.zip › Figure S8_Proofread.png]

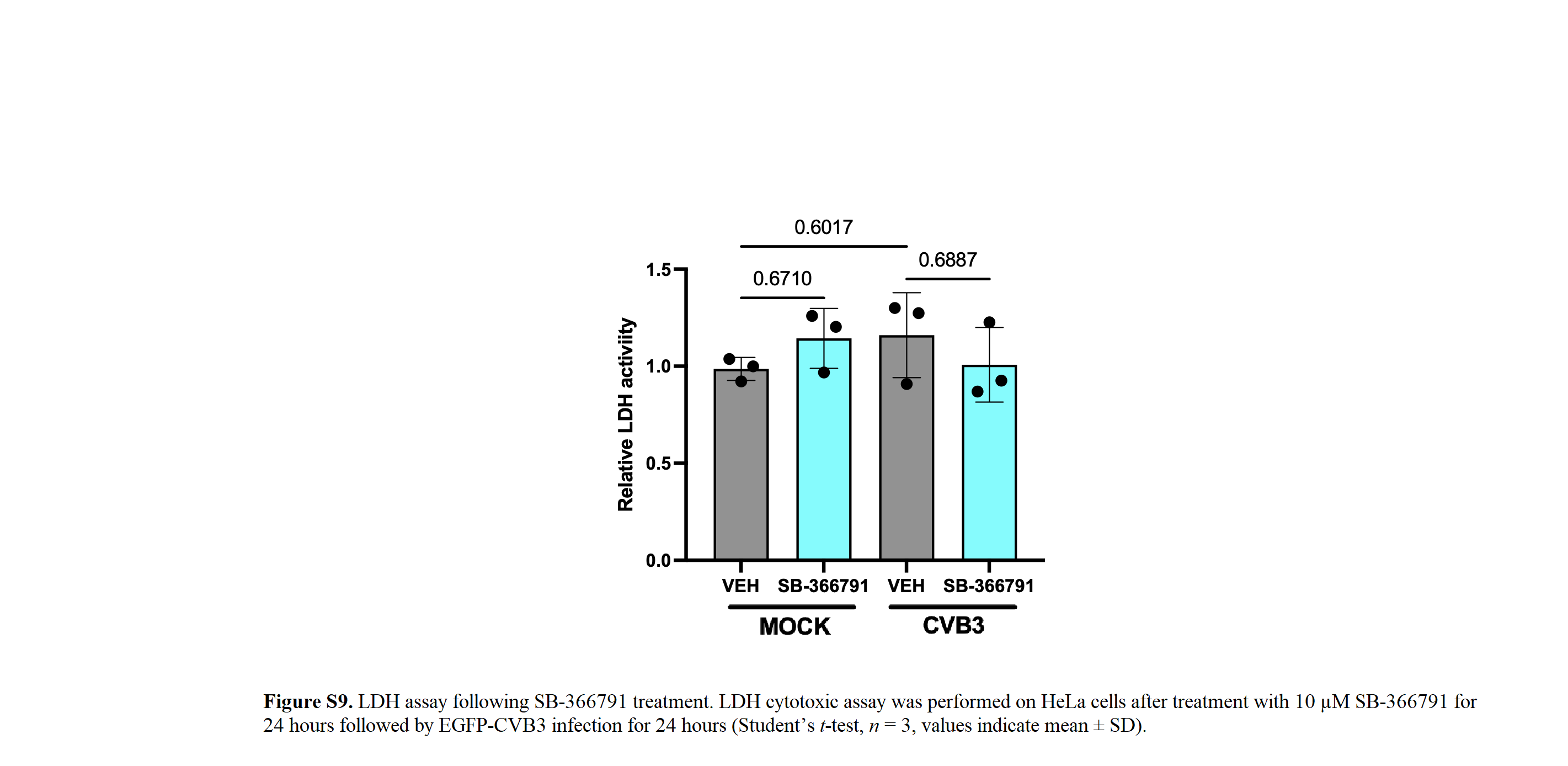

Supplement: Supplementary file 1 [file ijms-27-00661-s001.zip › Figure S9_IJMS.png]

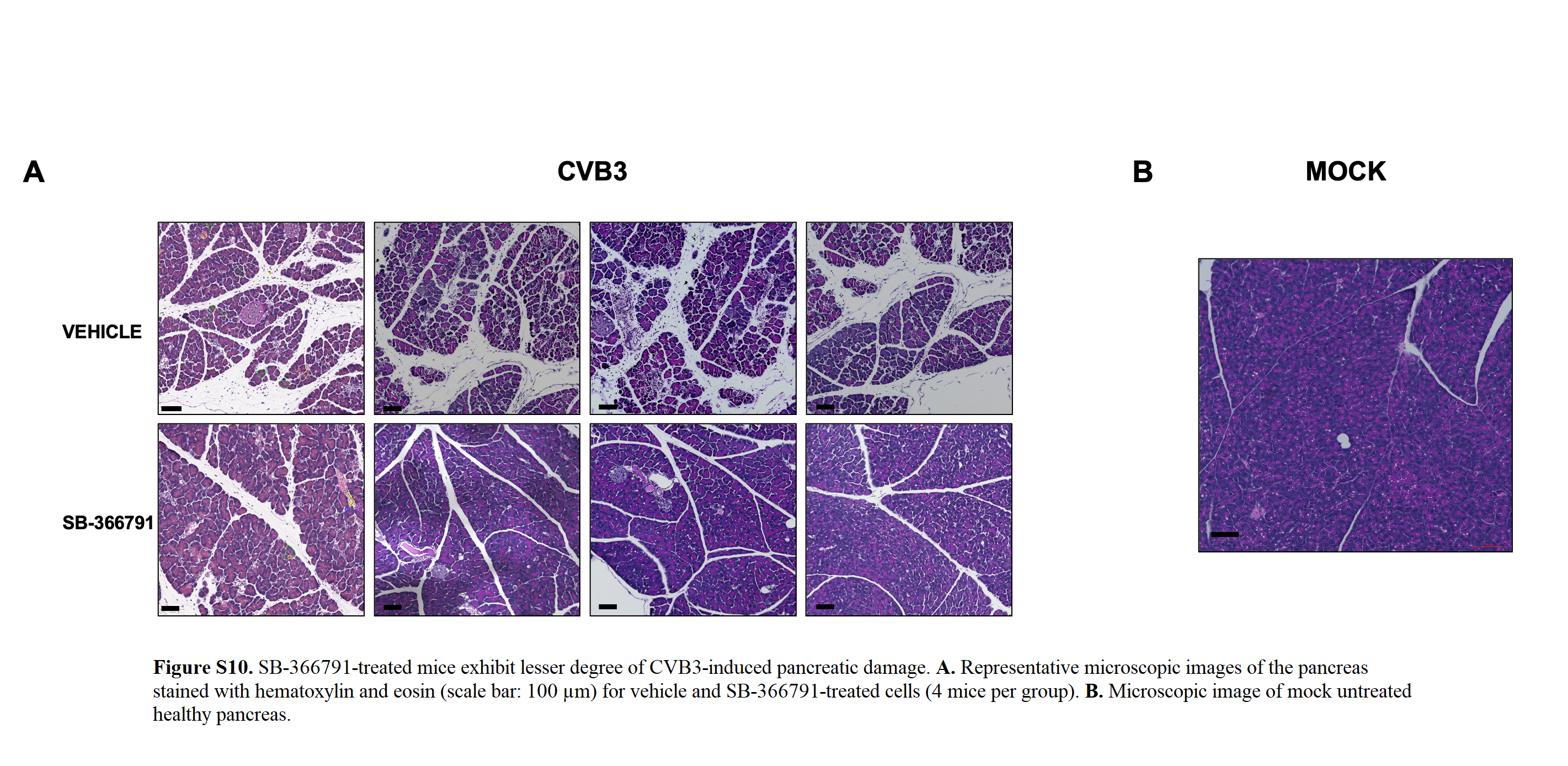

Supplement: Supplementary file 1 [file ijms-27-00661-s001.zip › Figure S10_IJMS.png]

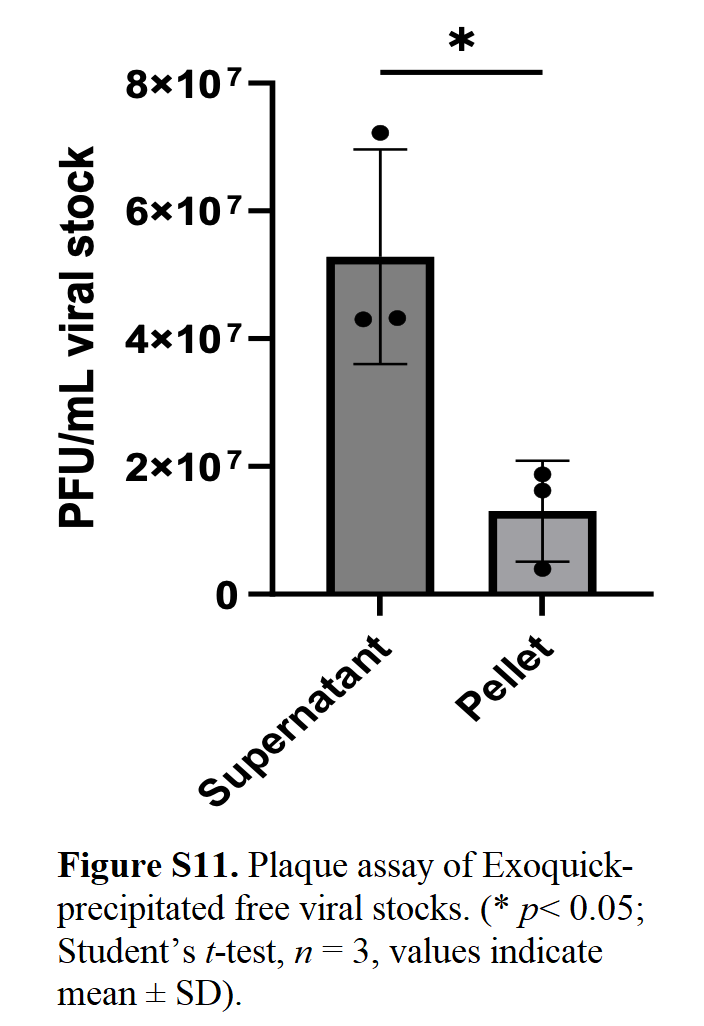

Supplement: Supplementary file 1 [file ijms-27-00661-s001.zip › Figure S11_IJMS.png]
